# An Ethnobotanical study of Medicinal Plants in high mountainous region of Chail valley (District Swat- Pakistan)

**DOI:** 10.1186/1746-4269-10-36

**Published:** 2014-04-16

**Authors:** Mushtaq Ahmad, Shazia Sultana, Syed Fazl-i-Hadi, Taibi ben Hadda, Sofia Rashid, Muhammad Zafar, Mir Ajab Khan, Muhammad Pukhtoon Zada Khan, Ghulam Yaseen

**Affiliations:** 1 Department of Plant Sciences, Quaid-i-Azam University, 45320 Islamabad, Pakistan; 2 School of Chemical Engineering, Universiti Sains Malaysia, 14300 Nibong Tebal, Penang, Malaysia; 3 University of Peshawar, Peshawar, Pakistan; 4 Materials Chemistry Laboratory, Faculty of Sciences, Mohammed First University, Oujda 60000, Morocco

**Keywords:** Geo-ethnographical, Ethnoflora, Khyber Pakhtunkhwa, Pharmacological studies

## Abstract

**Background:**

This paper represents the first ethnobotanical study in Chail valley of district Swat-Pakistan and provides significant information on medicinal plants use among the tribal people of the area. The aim of this study was to document the medicinal uses of local plants and to develop an ethnobotanical inventory of the species diversity.

**Methods:**

In present study, semi-structured interviews with 142 inhabitants (age range between 31–75 years) were conducted. Ethnobotanical data was analyzed using relative frequency of citation (RFC) to determine the well-known and most useful species in the area.

**Results:**

Current research work reports total of 50 plant species belonging to 48 genera of 35 families from Chail valley. *Origanum vulgare*, *Geranium wallichianum* and *Skimmia laureola* have the highest values of relative frequency of citation (RFC) and are widely known by the inhabitants of the valley. The majority of the documented plants were herbs (58%) followed by shrubs (28%), trees (12%) and then climbers (2%). The part of the plant most frequently used was the leaves (33%) followed by roots (17%), fruits (14%), whole plant (12%), rhizomes (9%), stems (6%), barks (5%) and seeds (4%). Decoction was the most common preparation method use in herbal recipes. The most frequently treated diseases in the valley were urinary disorders, skin infections, digestive disorders, asthma, jaundice, angina, chronic dysentery and diarrhea.

**Conclusion:**

This study contributes an ethnobotanical inventory of medicinal plants with their frequency of citations together with the part used, disease treated and methods of application among the tribal communities of Chail valley. The present survey has documented from this valley considerable indigenous knowledge about the local medicinal plants for treating number of common diseases that is ready to be further investigated for biological, pharmacological and toxicological screening. This study also provides some socio-economic aspects which are associated to the local tribal communities.

## Background

Wild resources of medicinal plants have been used by man for centuries in traditional healing systems. Indigenous people have adapted different modes of application and uses to exploit this natural resource [1]. The use of wild plants as food and medicine is prevalent in many rural communities of the world since times [2]. The local communities in many developing countries depend on plant based medicines even today, whereas, the modern system of health care is mainly dependent on plant based ingredients [3]. The traditional use of plants is inevitable in providing folk medicines for health care system and also as a source of food for the low income class and the rural communities. The indigenous system of folk medicines based on the use of plants by the local communities has been practiced for centuries and travels through generations from older to younger ones [4]. The use of plants in modern medicine has considerably increased, on the other hand traditional knowledge is gradually decreasing due to rapid urbanization and dependence of man on modern health care systems, but this folk system still prevails in the rural communities [5].

Pakistan has a rich floral diversity, represented by approximately 1572 genera and around 6000 wild plant species, which are mostly common in the Hindukush, Himalaya and Karakorum regions [6-8]. A number of representative studies have enumerated approximately 600 species of medicinal plants in the treatment of common ailments in the local communities of Pakistan [9]. Northern Pakistan is rich in terms of medicinal plants distribution and folk usage among the local tribal communities. Various ethnobotanical studies have been conducted in north-western parts of Pakistan and many have compiled information on the use of medicinal plants in other parts of the country [10-16]. Chail valley, district Swat has rich floral and cultural diversity due to its geographic location and prevailing climatic conditions. The valley has not been previously assessed ethnobotanically and in this regard the present study can be considered as the first of its kind. Wild medicinal plants, fruits and nuts add to the physical and cultural beauty of the valley.

The valley is bound by old custom and traditions and the inhabitants are mainly of low income class dependent on farming. Agriculture accounts for roughly 50% of the economic activities of valley and the major source of income for most of the rural population. The lack of communication with modern civilization has kept them closer to nature where they derive many of their day-to-day needs. The locals, in particular elders (men and women) and traditional healers (men), have centuries-old knowledge about the use of plants for treating range of common diseases [17].

The valley is under the effect of war on Terror since 9/11 due to its close vicinity with Afghanistan and the prevalent terrorist activities in the region, which has badly affected the socioeconomic conditions of the local inhabitants. The consequences of this war has led to huge disruption in both settled and tribal areas of Khyber Pakhtunkhwa province, particularly district Swat. Due to the militant activities, there is a heavy blow to the life of people in this area. A Preliminary Damage and Needs Assessment report prepared by the Asian Development Bank and the World Bank incorporates the issues of life losses, injuries, psycho-social losses, mass level migrations and internal displacement of the population in this region [18,19]. This war against terrorists and fighting in Swat is the first serious insurgent threat to the local communities in the area. Number of forces including some foreign fighters and some religious extremists are the causal agents for rapid decline of local population in small towns and villages of Chail valley for the last ten years [20]. These are the main threats in the valley that may lead in the extinction of indigenous knowledge regarding the use of medicinal plants in near future. On the other hand, the study area is far away from the urban setup and has specific rough mountainous geographic features, where there is a lack of government services as well as modern health care facilities. Considering all these issues generally and particularly the high percentage death rate of elderly population and fast migration of younger people from the area to other safer sites, it is felt worth to document the local knowledge of medicinal plant usage in the valley.

The aim of this work is to document the local traditions of medicinal plant use and encourage the preservation of this previously undocumented information for future generations. Data collected through field trips are compiled into an inventory exhibiting the plants reported and their frequency of citations together with the part used, diseases treated and the methods of application. In particular, we have compare the documented uses of traditional healing practices with previous ethnobotanical reports in our neighboring countries to evaluate the uniqueness of our current work in the region to provide new information on less reported medicinal plant species for future studies with global acceptance.

The region has peculiar social and economic setup which is mainly related to the floral and faunal diversity of the valley. The local people give much relevance to the wild plant species for their local traditional uses as medicine. Therefore an in-depth study is needed to record the indigenous knowledge and their socioeconomic impact (Additional file 1).

## Materials and methods

### Geo-ethnographical overview of the study area

**Figure 1:**
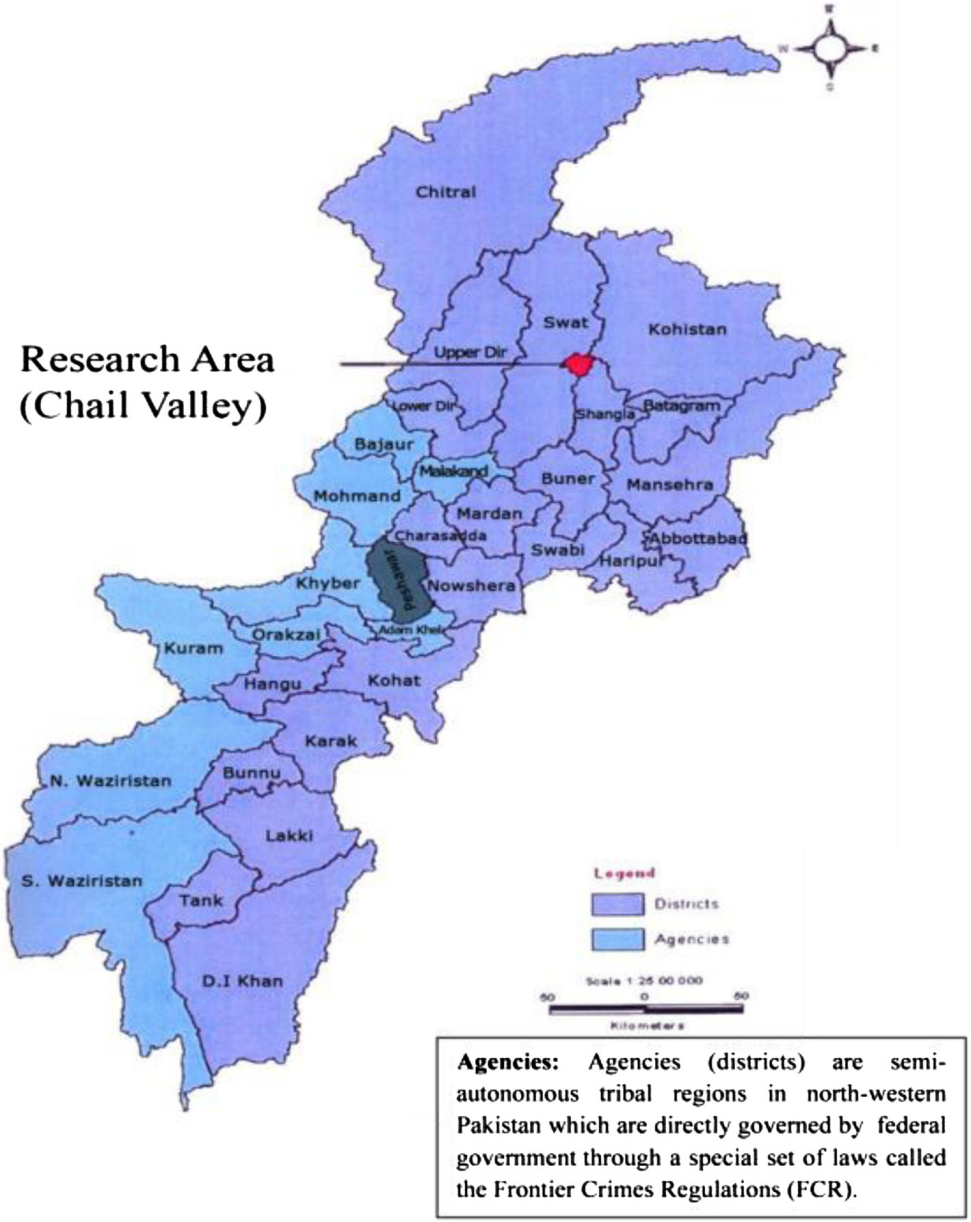
Geographical location of the study area.

The study was conducted in Chail valley, located in district Swat of Khyber Pakhtunkhwa province, Pakistan, near the border of Afghanistan and lies between 72°-36’ longitude and 35°-09’ N latitude. Topographically, the area is mountainous region situated in Hindukush foothills range. This range runs in the general direction of North and South with varied elevations starting from 1830 m above sea level up to 4270 m with total area of about 24,148 acres (Figure 1). The valley is home of lush green tracts, snow-covered glaciers, forests, meadows and plains. Geo-climatically the area falls within moist temperate zone where climate is controlled by various factors of latitude, altitude, summer monsoon and cyclonic current. Winter is extremely severe with coldest months of December to February and mean minimum recorded temperature is −2.4°C. Comparatively, summer, is fairly moderate with mean maximum recorded temperature is 36.32°C. The average annual rainfall and snowfall ranges from 500 mm to 1200 mm and 423.56 cm to 600 cm respectively [6,21].

The ethnic composition of the valley is quite diverse mainly resides with Pakhtuns (Yousafzai clan), kohistanis ajars and gujjars. Primary local language spoken in the area is Pashto (93% of the population) while the other locally known languages includes, torwali, kalami, khwar, kohistani and gujro (a mix of pashto and Punjabi) [22].

#### Socio economic conditions of Chail valley

Chail Valley Swat has a diversified flora with a large number of medicinal species, traditionally used as medicine and could be exploited for pharmaceutically and economically. The local community uses these plant resources as a medicine and also exploits it for monetary benefits, which is common practice since times. The topographical conditions along with severe cold weathers have a strong impact on the living of common people, but on the other hand act as a natural sanctuary for biodiversity. Rich biodiversity, indigenous system of living, faunal composition could possibly attract agro-forest industry; promote tourism and trade in the valley. This will strongly affect their living standards and have positive impact on the socioeconomic conditions of the local community.

#### Field interviews and data analysis

Ethnobotanical study was carried out to compile the indigenous knowledge about the use of medicinal plants for the treatment of ailments by the local communities of Chail valley Swat, from March 2011 to July 2013. Team members in the field work were Mushtaq Ahmad, Syed Fazl-i-Hadi, Sofia Rashid, Muhammad Pukhtoon Zada Khan and Ghulam Yaseen, who visited the study area in spring and summer seasons and a total of twelve field studies were conducted two each per season. Ethnobotanical information was collected from native inhabitants of the valley using semi-structured questionnaires [23]. Field work consisted of data documentation, plant collection and photography. Prior rural appraisal (PRA) approach was adopted according to Kyoto protocol concerning the intellectual property rights of local inhabitants and plant resources of the area. Formal written consent, including consent for publication was received from all the informants before the interviews began. The method employed during the study was designed with the sole purpose for eliciting the precious wealth of local knowledge on medicinal plant use. We interviewed 142 informants with aged range between 31–75 years in twelve different remote villages of valley. The informants were asked various questions about their traditional knowledge, plant use, disease treated, part used and the method of preparation and administration. Interviews were generally conducted in local language (Pashto) at village male gathering places (*Hujras*), mosques and sometime in houses. All documented data were then translated into English.

Field trips were conducted in spring and summer season, accompanied by local informants for identification and collection of plant species used in the valley. In most of the cases, the inhabitants helped in plant collection, showing their close association and familiarity with the area and flora. The rules followed during plant collection were according to the National biodiversity action plan for Pakistan [24] and dully signed from the Herbarium of Pakistan (ISL). The plants in this survey were classified into herbs, shrubs, trees and climbers using the Raunkiar’s (1934) life form classification system. The voucher specimens of collected plant species including their taxonomic and ecological information were prepared and authenticated using Flora of Pakistan [25] and then submitted to the Herbarium (ISL) of Quaid-i-Azam University Islamabad for future studies.

Data documented during the ethnobotanical survey was entered on a Microsoft excel database and analyzed to determine the proportions of different variables like gender and age wise percentage of folk medicinal knowledge, life form representation, plant parts used, preparation methods and most frequently treated diseases. Furthermore, we determined the Relative Frequency of Citation (RFC) of reported species using following index;

$$\begin{array}{cc} \mathrm{RFC}=\mathrm{FC}/N & \left(0<\mathrm{RFC}<1\right) \end{array}$$

This index shows the local importance of each species and it is given by the frequency of citation (FC, the number of informants mentioning the use of the species) divided by the total number of informants participating in the survey (N), without considering the use-categories [4,5,9].

## Results and discussion

### Demographic data and cultural background

**Figure 2:**
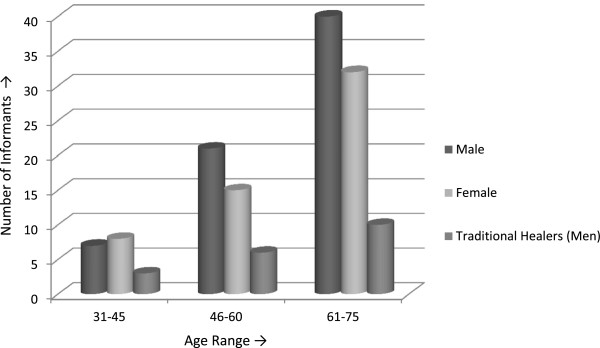
Distribution of gender, age and number of informants interviewed.

A total of 68 men (48%), 55 women (39%) and 19 men traditional healers (13%) were interviewed. The informants were divided into three age groups (1) 31–45, (2) 46–60 and (3) 61–75 years old. Most of the informants belonged to age between 61 and 75 years. Eighty two were aged between 61 and 75 years, 42 between 46 and 60 years and 18 between 31 and 45 year (Figure 2). In all twelve villages of the valley, ethno-botanical information was documented mainly from men and traditional healers during open discussions at male meeting sites (*Hujras*) and religious places (*Mosques & Madrasas*). Present study was unique in comparison to previous ethno-botanical surveys conducted in other parts of the country, as in this context, we interviewed the female community for the first time in the Chail valley of district Swat. In this area, the societal norms among the female members are very traditional and conservative, holding strong to the popular value system deriving its basic principles from their religion. The concepts of shame and honor, hospitality, gender segregation and veiling (*Parda*) are predominant within female community. Fine shades in division of labor are obvious in this area; women generally manage the domestic life, whereas the male members are responsible for earning and representing the family at communal level [1]. Furthermore, females are strictly not allowed to talk with male members of the community except close relatives. Ethnobotanical information from female members of the community was collected with the help of old ladies (Mah Je). Interviews with female informants were held at their houses and girl’s schools. In addition to this, the purpose of data collection from female informants was to compare their indigenous knowledge with male members and also to know their interest regarding the use of medicinal plants to treat different ailments. Generally, the semi-structured questionnaire based interviews with local inhabitants began after explaining the purpose of this survey to the subject. In order to collect detailed information relating to herbal medicine, inhabitants of the community were requested to share the knowledge of medicinal plants utilization in local language. It can be assumed from this study, that all members of the community generally relied on traditional herbal medicines due to the un-availability of modern heath care facility in the valley. Similarly, easy availability and rich diversity of medicinal plants in the area may also influence the decision regarding the use of herbal medicines by the local people.

It is noted during the survey, that the female informants in comparison to male members have a significant knowledge about the preparation and administration of herbal drugs which reflect their role in house hold management and disease treatment in order to keep the family healthy. While, their role as a plant collector particularly in rough and steep mountainous tracts of the area was found to be less as compared to men and traditional healers. Similar observations were made during the ethnobotanical survey conducted in Batan, Island, Philippines by Abe and Ohtani [26], who also found that the women play an important role in preparation of traditional medicines using medicinal plants. However, the diagnostic techniques to cure common ailments were often very primitive in the valley as similar to the Unani and Islamic (Tibb) systems of medicine. Diseases like diarrhea, dysentery, skin infections, snake bites, dysmenorrhea (Period pain among women) and rheumatism were obviously easily diagnosed by the elder men, women and traditional healers in the area. The color of the tongue, eyes, cold (*Sard*) and hot (*Garam*) conditions of the body were common indicators to be used to understand the patients’ problem.

### Medicinal flora

**Figure 3:**
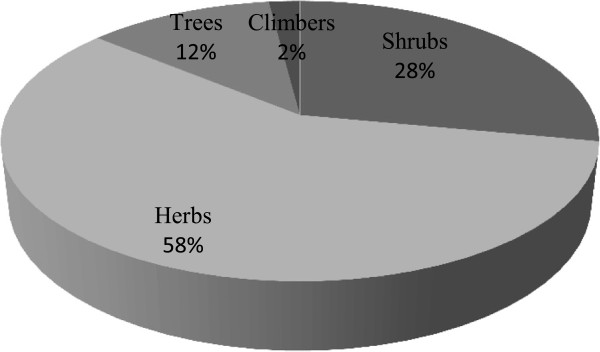
Life form representations of medicinal plants.

During the fieldwork of the present study, we collected data on 50 species belonging to 48 genera of 35 flowering plant families which have medicinal use (Table 1). The complete inventory of the ethnoflora consisting of taxon name in alphabetical order with voucher specimen number, family name, local name followed by life form, part used, preparation method, disease treated, phytochemical constituents, frequency citation (FC) and relative frequency of citation (RFC). The best represented used families in terms of the number of species were Lamiaceae (six species) and Polygonaceae (three species), while Amaranthaceae, Apiaceae, Ranunculaceaes, Rhamnaceae, Rosaceae, Rutaceae, Scrophulariaceae and Solanaceae were represented with two species each and other 25 families with one species each. The values and characteristics of Lamiaceae family as predominant in this area are similar to those of previous studied ethnoflora [27-30]. However, the predominance of Polygonaceae in our survey agreed with this statement that the more common a plant taxon in an area, the greater the probability of its popular use. The reported members of Polygonaceae in Chail valley includes *Bistorta amplexicaule, Rheum australe* and *Rumex hastatus,* those are commonly use as edible wild vegetables and to cure abdominal ailments, diarrhea and constipation by the local people. In terms of the life form, the highest number (29) of species used were herbaceous habit followed by shrubs (14), trees (6) and then climbers (1) (Figure 3). This is not surprising, that the herbaceous habit is dominant life form in our study but it is a common and widespread ecological phenomenon around the world [17,31]. The majority of documented plants in the valley are distributed generally in hilly tracts, near water bodies and in waste places as wild. However, a few common used species were found to be cultivated on small scale by the local farmers and household ladies for their use by themselves, for relatives and neighbors but not for marketing purpose. *Mentha longifolia, Swertia chirayita, Plantago lanceolata, Origanum vulgare, Viola pilosa, Zanthoxylum armatum* and *Zizyphus jujuba* were some common cultivated species in the valley*.* These species are used in the form of herbal teas, spices and powder drugs for the cure of ailments due to their rich and fragrant flavors. It is considered that understanding of the market potential for medicinal plants could provide rural farmers with the incentive for cultivation of high demand species in future.

**Table 1:** Medicinal plants use in Chail valley

*Taxon (BN/ VSN/ F/LN)	**Life form	**Part used**	**Preparation method**	**Disease treated**	***FC n = 142	****RFC	*** Previous uses in Neighboring regions
*Achyranthes aspera* L.	H	Stem	Dried ground stem mixed with powdered leaves of *Menthalongifolia* and taken orally.	High Fever, Chest problems	49	0.34	1 ●, 2 ▲, 3 ●, 4 ▲
ISL 582							
Amaranthaceae							
Spay Butay							
*Aconitum chasmanthum* Stapf ex Holmes	H	Rhizome	Powder of dried rhizomes is used.	Joint pain, High fever, Stupor, Soothing	111	0.78	1 ●, 2 ●, 3 ●, 4 ●
ISL 614							
Ranunculaceae							
Zahar Mora							
*Aconitum heterophyllum* Wall. ex Royle	H	Roots and rhizomes	The dried pulverized roots are mixed with butter oil and taken orally.	Mild fever, Sexual weakness, Anthelminthic	69	0.48	1 ●, 2 ●, 3 ●, 4 ●
ISL 615							
Ranunculaceae							
SarbaZelai							
*Acorus calamus* L.	H	Root	Dried powdered roots are mixed with sugar and taken orally.	Digestive disorders, Chronic dysentery	43	0.30	1 ●, 2 ●, 3 ■, 4 ■
ISL 587							
Aracaceae							
Skhawaja							
*Ajuga bracteosa* Wall. ex Benth	H	Leaves	Leaves are washed, boiled in water and left overnight in dew; this decoction is taken before breakfast.	Skin infections, High fever	35	0.24	1 ■, 2 ●, 3 ●, 4 ●
ISL 599							
Lamiaceae							
Khawaja booti							
*Amaranthus spinosus* L.	H	Leaves and young shoots	Leaves and young shoots are boiled in water and this decoction is directly used.	Biliousness	45	0.31	1 ▲, 2 ●, 3 ▲, 4 ●
ISL 583							
Amaranthaceae							
Chalveray							
*Ammi visnaga* (L.) Lam.	H	Fruit and flower	Dried fruits and flowers mixed with dried fruits of*Buniumpersicum*, ground and taken.	Heart diseases, Bronchial asthma/Breathing problems, Whooping cough	107	0.75	1 ●, 2 ●, 3 ●, 4 ●
ISL 585							
Apiaceae							
Sperkai, Gangahai							
*Artemisia biennis* Willd.	H	Leaves	Leaves are boiled in water and decoction is taken, Warm poultice of leaves is applied externally.	Digestive disorders, Anthelminthic, Skin infections	27	0.19	1 ●, 2 ●, 3 ●, 4 ●
ISL 590							
Asteraceae							
Tarkha							
*Atropa acuminate* Royle ex Lindl.	H	Leaves, roots and floral buds	Powdered roots, leaves and floral parts are taken with milk twice a day.	Urinary disorders, Stupor, Asthma/breathing problems	32	0.22	1 ●, 2 ●, 3 ●, 4 ●
ISL 626							
Solanaceae							
Bargak							
*Berberis lyceum* Royle	S	Root	Roots are dried and ground, butter oil is mixed with powder drug and taken with milk.	Hepatitis, Menorrhagia, Chronic fever, Jaundice	127	0.89	1 ●, 2 ●, 3 ●, 4 ■
ISL 591							
Berberidaceae							
Speen Kwaray							
*Bergenia ciliata* (Haw.) Sternb.	H	Rhizome	Decoction of rhizome is taken thrice a day.	Urinary disorders, Skin infections, Demulcent	44	0.30	1 ●, 2 ●, 3 ●, 4 ■
ISL 623							
Saxifragaceae							
Gat panra,							
*Bistorta amplexicaulis* (D.Don) Greene	H	Roots	Powdered roots are mixed with sugar and used with milk.	Urinary disorders, Cough, Sore throat, Joint pain	65	0.45	1 ●, 2 ●, 3 ●, 4 ●
ISL 611							
Polygonaceae							
Anjabar, Tarvapanra							
*Bunium persicum* (Boiss.) B. Fedtsch	H	Fruits and leaves	Fresh ground leaves are taken orally, Fruits are boiled in water and decoction is used.	Hearts problems	17	0.11	1 ●, 2 ●, 3 ●, 4 ●
ISL 586							
Apiaceae							
Thorazera, Da ghrasperkai							
*Calotropis procera* (Aiton) Dryand	S	Stem, leaves and flowers	Decoction of stem and leaves is taken; Latex is mixed with castor oil and applied on skin.	Malarial fever, Ulcer, Eczema, Ring worms	36	0.25	1 ●, 2 ●, 3 ●, 4 ▲
ISL 589							
Asclepiadaceae							
Spulmai							
*Duchesnea indica* (Andrews) Focke	H	Leaves	Black pepper is mixed with decoction of leaves and taken in morning for regulating mental disorders.	Mental disorders, Sexual weakness	23	0.16	1 ●, 2 ●, 3 ●, 4 ●
ISL 618							
Rosaceae							
Da zamakay toot							
*Elaeagnus angustifolia* L.	S	Whole plant	The paste of young shoots is mixed with juice of *Datis cacannabina* and applied on forehead for curing headache.	Headache, Heart burning, Skin infections	113	0.79	1 ●, 2 ●, 3 ●, 4 ●
ISL 593							
Elaeagnaceae							
Ghanum Rangay							
*Fragaria nubicola* (Hook. f.) Lindl. ex Lacaita	H	Leaves and roots	Leaves and roots in powdered form are mixed *Berberis lycium* and used directly.	Skin infections, Urinary disorders, Diarrhea	41	0.28	1 ●, 2 ●, 3 ■, 4 ●
ISL 619							
Rosaceae							
Da Zmakay Toot							
*Fumaria indica* (Hausskn.) Pugsley	H	Whole plant	Decoction of plant is used thrice a day by adults.	Blood purifier, High fever, Chest pain	61	0.42	1 ●, 2 ●, 3 ●, 4 ●
ISL 596							
Fumariaceae							
Paprra							
*Geranium wallichianum* D. Don ex Sweet	H	Rhizomes	Rhizomes are dried and ground into powder form. This powder is mixed in wheat flour, sugar, butter oil and traditional sweet dish is cooked and is taken once in a day.	Backache, Mouth ulceration, Chronic diarrhea	132	0.92	1 ●, 2 ●, 3 ●, 4 ●
ISL 598							
Geraniaceae							
Srazela, Ratanjot							
*Hedera nepalensis* K. Koch	H	Leaves	Fresh and ground leaves are boiled in water and taken orally.	Heart disease, Cancer, Diabetes	105	0.73	1 ●, 2 ●, 3 ●, 4 ●
ISL 588							
Araliaceae							
Palulzelai							
*Isodon rugosus* (Wall. ex Benth.) Codd	S	Shoots and seeds	Seeds are boiled in water and then this decoction is taken orally.	Skin infections, blood purifier, Gastric and abdominal pain	18	0.12	1 ●, 2 ●, 3 ●, 4 ●
			Fresh shoots are crushed and soup is made which is taken orally.				
ISL 600							
Lamiaceae							
Sperkai, krachai							
*Justicia adhatoda* L.	S	Root and leaves	Dried, ground leaves mixed with sugar and root extract mixed with honey are taken orally.	Asthma, Cold, Cough, High fever	86	0.60	1 ●, 2 ▲, 3 ●, 4 ■
ISL 581							
Acanthaceae							
Bhaikar							
*Maytenus royleana* (Wall. ex M.A. Lawson) Cufod	S	Whole plant	Fresh leaves are collected, cleaned, crushed and extract is used as eyes drops.	Toothache, Eyes inflammation	45	0.31	1 ●, 2 ●, 3 ●, 4 ●
ISL 592							
Celastraceae							
Spin Azghkay							
*Melia azedarach* L.	T	Fruit	Ripened fruits are crushed and mixed with wheat flour.	Leprosy, Urinary disorders	49	0.34	1 ●, 2 ●, 3 ●, 4 ●
ISL 605							
Meliaceae							
Shandai							
*Mentha longifolia* (L.) L.	H	Leaves, shoots and floral tops	Dried leaves are ground to form powder drug and then taken orally.	Joint pain, Digestive disorders	126	0.88	1 ■, 2 ●, 3 ●, 4 ▲
ISL 601							
			Leaves are boiled in water and then taken for curing digestive disorders.				
Lamiaceae							
Velanay							
*Micromeria biflora* (Buch.-Ham ex D. Don) Benth.	H	Stem and leaves	Stem and leaves are chewed and juice swallowed.	Urinary disorders, Digestive disorders	71	0.5	1 ●, 2 ●, 3 ●, 4 ●
ISL 602							
Lamiaceae							
Naraishamakai							
*Olea ferruginea* (Sol.) Steud.	T	Leaves	Leaves are boiled in water and decoction is used thrice a day.	Gonorrhoea, High fever, Skin infections	65	0.45	1 ●, 2 ●, 3 ●, 4 ●
ISL 606							
Oleaceae							
Khona							
*Origanum vulgare* L.	H	Whole plant	Decoction of plant is taken orally.	Skin infections, Sexual weakness, Digestive disorders, Intestinal pain, Urinary disorders	135	0.95	1 ●, 2 ●, 3 ●, 4 ●
			Juice made from crushed plant is used for curing stomach and urinary pain.				
ISL 603							
Lamiaceae							
Shamakai							
*Otostegia limbata* (Benth.) Boiss.	S	Leaves	Leaves are dried, ground and powder is mixed with honey. A table spoon is taken once a day for wound healing.	Gum disease, Wounds	73	0.51	1 ●, 2 ●, 3 ●, 4 ●
ISL 604							
Lamiaceae							
SpeenAzghakay							
*Oxalis corniculata* L.	H	Whole Plant	Juice of fresh plant is used in stomach problems.	Digestive disorders, wounds,	46	0.32	1 ●, 2 ●, 3 ●, 4 ●
ISL 607			Fresh leaves are crushed and applied to wounds to stop bleeding.	Jaundice,			
Oxalidaceae							
ManzakeenTarukay							
*Paeonia emodi* Royle	H	Roots	Dried ground roots are fried and taken orally.	Diarrhea, Rheumatic pain, Gynecological disorders, Vomiting	92	0.64	1 ●, 2 ●, 3 ●, 4 ●
ISL 608			Powdered roots mixed with butter oil and taken orally.				
Paeoniaceae							
Mamaikh							
*Picrorhiza kurrooa* Royle.	H	Roots and rhizomes	Decoction of rhizome is taken twice a day for a week.	Digestive complaints, Heart disease	29	0.20	1 ●, 2 ●, 3 ●, 4 ●
ISL 624							
Scrophulariaceae							
Karroo							
*stacia integerrima* J.L. Stewart ex Brandis	T	Bark and fruit	The boil extract of bark is used.	Jaundice, Bronchial disorder	66	0.46	1 ●, 2 ●, 3 ●, 4 ●
ISL 584			Fruit is roasted in mustard oil, ground and then used.				
Anacardiaceae							
Shanai, Kakarsinghai							
*Plantago lanceolata* L.	H	Leaves and fruits	Leaves and fruits are boiled in water. The extract is cooled and mixed with sugar which is used for curing dysentery.	Urinary disorders, dysentery, Skin sores, Burns, Wounds	112	0.78	1 ●, 2 ●, 3 ▲, 4 ●
ISL 609							
Plantaginaceae							
BaltangaJabai							
*Podophyllum emodi* Wall. ex Royle	H	Roots	A decoction of dried ground roots is taken thrice a day.	Vomiting, Purgative, Abdominal pain	57	0.40	1 ●, 2 ●, 3 ●, 4 ●
ISL 610							
Podophyllaceae							
Gangora, Kakorra							
*Quercus incana* Bartram	T	Bark and fruits	Half roasted fruit powder is mixed in honey and taken.	Urinary disorders, Asthma/breathing problems, Diarrhea, Gonorrhoea	50	0.35	1 ●, 2 ●, 3 ●, 4 ●
ISL 595							
Fagaceae							
Spin Banj							
*Rheum australe* D. Don	H	Whole plant	Powder of plant is sprayed over the wounds for early healing.	Intestinal pain, Skin infections, blood purifier	71	0.5	1 ●, 2 ●, 3 ●, 4 ●
ISL 613							
Polygonaceae							
Chontal							
*Ricinus communis* L.	S	Leaves and seeds	Leaves are heated and applied over the abdomen.	Constipation, Jaundice, Abdominal pain	64	0.45	1 ●, 2 ●, 3 ●, 4 ●
ISL 594							
Euphorbiaceae							
Aranda, Harhanda							
*Rumex hastatus* D. Don	S	Leaves and young shoots	Decoction of leaves and young shoots is taken thrice a day for the treatment of constipation	Urinary disorders, Cooling, Constipation, Worms problem	81	0.57	1 ●, 2 ●, 3 ●, 4 ●
ISL 612							
Polygonaceae			Dried ground leaves are taken orally with water for removal of tape worms.				
Tarukay							
*Salix babylonica* L.	T	Leaves	Leaves are warmed and wrapped around the infected wounds and areas of insect bites for releasing their poisonous effects.	Wounds, Insect bites	69	0.48	1 ●, 2 ●, 3 ●, 4 ●
ISL 622							
Salicaceae							
Aseelawala							
*Skimmia laureola* (DC.) Siebold & Zucc. exWalp.	S	Leaves	Dried ground leaves are taken orally with water.	Small pox, Worms problems, colic	129	0.90	1 ● , 2 ● , 3 ● , 4 ●
ISL 620							
Rutaceae							
Nazarpanra, Nameer							
*Swertia chirayita* Roxb. H. Karst.	H	Whole plant	An infusion of the herb is generally employed.	Digestive disorders, Hepatitis, Worms problems, Dyspepsia, Diarrhea	82	0.57	1 ▲, 2 ●, 3 ●, 4 ■
ISL 597							
Gentianaceae							
Chirayata							
*Valeriana jatamansi* Jones	H	Rhizome	Dried ground rhizome is taken orally with milk.	Intestinal pain, neurosis, Insomnia, Constipation	61	0.42	1 ●, 2 ●, 3 ▲, 4 ■
ISL 628							
Valerianaceae							
Shingatai							
*Verbascum thapsus* L.	H	Leaves, floral parts and seeds	Decoction of seeds is taken orally.	Diarrhea, Dysentery, Analgesic, Skin infections	42	0.29	1 ●, 2 ●, 3 ●, 4 ●
			Decoction of floral part is applied externally to the burnt part of the body.				
ISL 625							
Scrophulariaceae							
KharGhwag							
*Viola pilosa* Blume	H	Leaves and flowers	Decoction of dried ground leaves and flowers mixed with honey is used thrice a day.	Chest pain, High and mild fever, Stomach ulcer	115	0.80	1 ●, 2 ●, 3 ●, 4 ●
ISL 630							
Violaceae							
Banafsha							
*Vitex negundo* L.	S	Leaves and stem	Leaves are boiled in water, filtered and extract is use orally.	Mild fever, Urinary disorders, Worms problem anthelmintic	78	0.54	1 ●, 2 ●, 3 ●, 4 ●
ISL 629							
Verbenaceae							
Marwandai							
*Withanias omnifera* (L.) Dunal	S	Whole plant	Roots are ground and mixed with wheat flour and butter oil and a traditional sweet is made which is used as tonic.	Urinary disorders, Sexual weakness, narcotic, Rheumatic pain	83	0.58	1 ●, 2 ■, 3 ●, 4 ●
ISL 627							
Solanaceae							
Kutilal							
*Zanthoxylum armatum* DC.	S	Bark and fruit	Decoction of bark is use orally.	Digestive disorders, Skin infections, Gum diseases, Toothache	123	0.86	1 ●, 2 ●, 3 ●, 4 ■
ISL 621			Young shoots are chewed and juice is swallowed to treat the gum diseases and toothache.				
Rutaceae							
Dambara, Timbar							
*Zizyphus jujuba* Mill.	T	Leaves and fruits	Leaves are chewed by diabetic patients to decrease sugar level.	Skin infections, Diabetes	112	0.78	1 ▲, 2 ●, 3 ●, 4 ▲
ISL 616							
Rhamnaceae							
Markhanai, Unnab							
*Zizyphus oxyphylla* Edgew.	S	Roots and fruits	Roots are crushed and boiled in water. The extract is cooled, filtered and kept in dew overnight. This decoction is taken in morning for curing jaundice.	Jaundice, Burnings	79	0.55	1 ▲, 2 ●, 3 ●, 4 ▲
ISL 617							
Rhamnaceae							
Markhanai, Elanai							

*BN = Botanical Name, VSN = Voucher Specimen Number, F = Family, VN = Vernacular Name.

**H = Herb, S = Shrub, T = Tree.

***FC = Frequency Citation, ****RFC = Relative frequency of Citation.

Symbols indicate comparison of use with neighboring regions: (■) Similar use, (▲) Different use. (●) Use not reported.

It is noted during present study that both male and female members in this valley are the main users of plants for treating their common ailments. However, some plants were specific to men as sexual tonic (e.g. *Aconitum heterophyllum*) and certain plants are only given to women for gynecological disorders (e.g. *Paeonia emodi*). Furthermore, the elders above 60 years and small children below 10 years may also give particular plants suited to their specific age limiting ailments. Of all medicinal plants reported in the valley, *Origanum vulgare*, *Geranium wallichianum* and *Skimmia laureola* seems to be the most well-known to the locals as indicated by their high RFC values. However, our these results are in contrast to somewhat with previous studies, where different plant species were reported with respect to their preference use [14,21,23,32-34]. The other most cited medicinal species in our survey includes *Berberis lycium*, *Mentha longifolia*, *Zanthoxylum armatum*, *Viola pilosa*, *Elaeagnus angustifolia*, *Plantago lanceolata*, *Zizyphus jujuba*, *Ammi visnaga* and *Hedera nepalensis*.

The degree of ethnobotanical richness in the valley is mainly due to its rich diversity of medicinal plants with traditional uses by local tribal communities. In addition to this, the RFC values also indicate the importance of species relative to the number of local informants taking part in this study. This reflects the strong and long term association of inhabitants with local plants. However, our reported results regarding most frequently used family, species and disease treated using medicinal plants in this are considerably varied from other parts of the world [35,36].

### Herbal drug preparation and utilization

**Figure 4:**
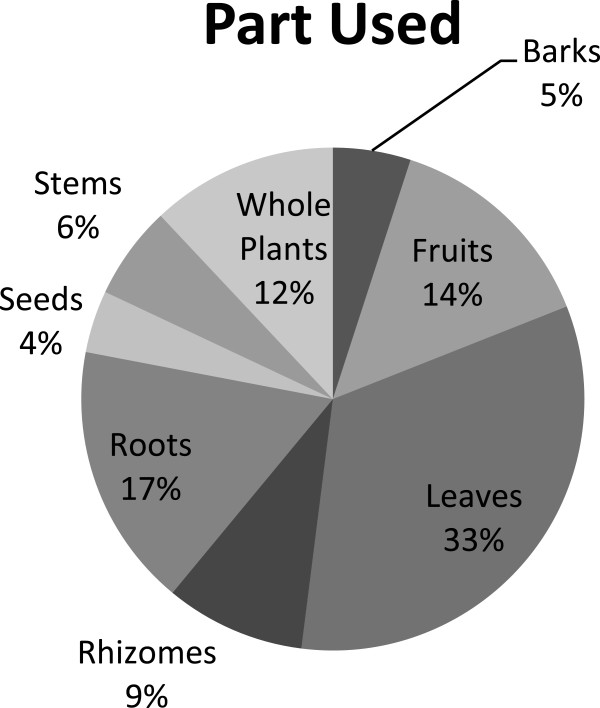
Frequency of plant parts used for medicinal applications.

In the present survey, we found that almost all parts of the different species were used against common diseases. The most commonly used plant parts in herbal preparations were leaves (33%), followed by roots (17%), fruits (14%), whole plants (12%), rhizomes (9%), stems (6%), barks (5%), and seeds (4%) (Figure 4).In many cases, more than one part of the same species, generally leaves and aerial parts (comprising stems, branches and flowers) are used in different herbal preparation and remedies. Leaves as frequently used organ in traditional herbal drugs is also reported in previous ethnobotanical studies [25,26,37]. In addition to this, leaves are the main photosynthetic organs in plants and are considered to be the natural pharmacy for synthesis of many active constituents those are pharmacologically more active against certain diseases [38]. With similar to previous reports, it is also noted in this work that roots are frequently used part, second to leaves [39-42]. The utilization of fruit by locals in Chail valley after leaves and roots is found to be widespread as compare to the whole plant use. This agrees with Rashid et al. [41] who also reported the rich diversity of edible wild fruits with medicinal uses in Swat region of northern Pakistan.

**Figure 5:**
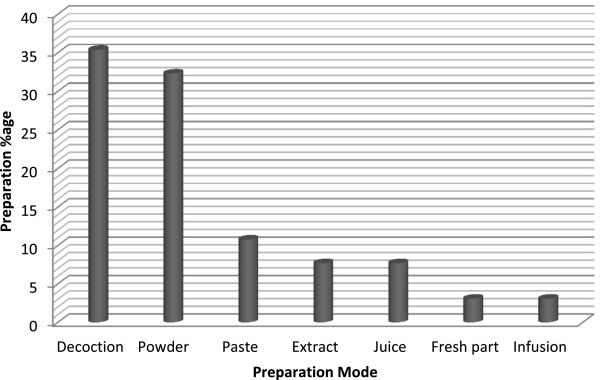
Frequency of herbal drug preparation methods.

**Figure 6:**
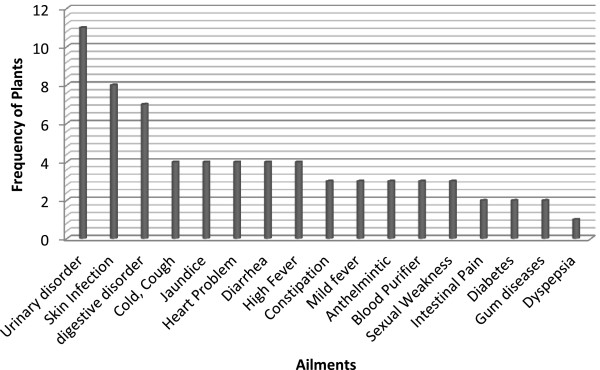
Most frequently treated ailments using medicinal plants.

The most important forms of preparation methods for herbal drugs were decoction (23 records) followed by powder drug (21 records), paste (7 records), extract (with oil/milk) (5), juice (5) and fresh part (2) and infusion (2 records) (Figure 5). The dosage of the medicinal preparation (quantity, doses, frequency, period of use, etc.) is not very precise, as it generally varied based on application, disease, age, patients physical health, illness severity, diagnosis and experience of traditional healer. It is reported that almost all of the documented species use singly as mono-herbal recipes with their specific part use for particular disease while some timesthe mixtures of various parts (e.g. aerial parts) with additional ingredients like milk, honey or butter may be used to treat some diseases. It was also observed in few cases; preparations consist of more than one plant species in different combinations to treat same disease. It is reported that total of 56 different ailments were treated in the valley using medicinal plants. Figure 6 shows that the maximum number of species were used for urinary disorders (11 species) followed by skin infections (8 species) and digestive disorders (7 species). These predominant disorders were also reported in the valley by the health department of Khyber Pakhtunkhwa province and Pakistan Demographic and Health survey (PDHS) [43]. The main reason for the use of medicinal plants by the inhabitants of the valley is the lack of basic health units, rich diversity of medicinal plants and the area is far away from the city.

### Novelty index

The present study was comparatively analyzed within the region as well as with aligned countries. Published research papers were selected randomly. In Pakistan all studies published from 2008–2013 were analyzed while from aligned countries 9 research papers from 2000–2013 were reviewed. It was observed that majority of plants reported in the present study are confined to the present geographical region because the study area is linked with lesser Himalayas and may be due to their native habitats and supporting conditions. Some plants of this study area were also widely distributed in aligned countries due to their wide adaptability in different ecological zones and reported for medicinal uses but during comparative analysis some new medicinal uses were observed. In aligned countries *Achranthes asper, Aconitum heterophyllum, Ammi visnaga, Berginia ciliate* were also reported but their uses vary from our study except a few. *Aconitum chasmanthum, Isodon rugosus* and *Micromeria biflora* were recorded for the first time from Pakistan as well as from aligned regions. Besides this, *Riccinus communis* was reported for the first time for jaundice and *Atropa acuminate* was reported for asthma.

Among all medicinal plants reported in Chail valley, *Origanum vulgare* L, *Geranium wallichianum* D. Don and *Skimmia laureola* (DC.) Sieb seems to be the most well-known to the locals as indicated by their high RFC values. While, our these findings are in contrast to previous ethnobotanical studies in neighboring countries as well as to other parts of the world, where different plant species were reported with respect to their preference use [6,36], [26], [34]. The other most cited medicinal plants based on RFC data includes *Berberis lyceum* Royle, *Mentha longifolia* L., *Zanthoxylum aromatum* Dc., *Viola pilosa* Blume, *Elaegnus angustifolia* L., *Plantago lanceolata* L., *Zizyphus jujuba.*

The current present study was quantitatively compared with four published research articles from aligned countries. Quantitative data between the medicinal plants uses in Chail valley and previous reports in the four selected neighboring countries were analyzed (Table 2).

**Table 2:** Comparative Quantitative data between Chail valley and four aligned countries based on medicinal uses

**Author (Year of study)**	**Area (Country)**	**Total No. of species reported**	**No. of species with similar uses as in Chail Valley**	**No. of Species with different uses from Chail valley**	**No. of species with new uses reported in Chail valley**	**Percentage of new uses reported in Chail Valley (%)**
Bhellum and Singh (2012)	District samba (Kashmir)	51	02	04	44	88
Sankaranarayanan, et al. (2010)	Villupuram district of Tamil nadu India	46	01	02	47	94
Long and Li (2003)	Jingping, Yunnan Province (China)	66	02	03	45	90
Kunwar et al. (2009)	Far west Nepal (Nepal)	135	07	05	38	76
Average Mean value	-	-	3	3.5	43.5	87%

### Future impact of the study

The study will provide a sense of social and economic responsibility among the common people, conserving the local flora. This valued information will also motivate the local community to attract tourism in the valley by preserving its natural beauty, which will enhance the socioeconomic prosperity and wellbeing of the rural community. The participation of the local community will help in conserving the floral diversity, promote trade and tourism. But meanwhile experts engaged in the policy making could possibly address the issues relate to the floristic composition and conservation. Besides the pharmaceutical and food industry could invariably exploit the local medicinal flora, which could be used for the public health and socioeconomic uplift of the region.

## Conclusion

This study contributed to the establishment of an inventory of plant based medicines used in Chail valley of District Swat-Pakistan. A total of 142 informants were interviewed during the survey to document the indigenous knowledge about the uses of medicinal plants. The present paper summarizes a data on 50 plant species used to treat 56 common ailments.RFC values ranked *Origanumvulgare* ,*Geranium wallichianum* and *Skimmialaureola* astop most cited and well known species in the valley. The majority of the plants were employed to treat diseases of urinary problems followed by skin infections, digestive disorders and diarrhea.The data provided by our tribal informants and analyzed in this paper clearly show that indigenous knowledge on medicinal plants uses is still alive in the Chail valley. The significance of this rich ethnopharmacological knowledge has furnished us with novel information that not only will provide recognition of this undocumented knowledge but also could provide basis for new avenues in future pharmacological screening that leads to natural drugs discovery development to improve healthcare systems globally. However to validate such information, detailed pharmacological studies must be carried out to improve the use of medicinal plants at global perspectives.This study also provides basis for the conservation of the local flora, its use as food and medicine. It will also provide various socioeconomic dimensions associated with the common people.

## Competing interests

The authors declare that they have no competing interests.

## Authors’ contributions

This work is part of an ethnobotanical field study carried out in high mountainous region of Chail-Valley (Swat), Pakistan.The manuscript is written by MA. SS and SR help in illustrations and drafting of manuscript. MA. SFH.SR. MPZK and GY.collected data in the field, MZ help in plant identification. TBH and MAK provide technical expertise in compiling data in to the manuscript. All the authors read and approved the final manuscript.

## Supplementary Material

Additional file 1 Graphical abstract of Chail Valley. Click here for file
