# Supplementary figures and images for: An Ethnobotanical study of Medicinal Plants in high mountainous region of Chail valley (District Swat- Pakistan)
Source: J Ethnobiol Ethnomed. 2014 Apr 16;10:36. doi: 10.1186/1746-4269-10-36 (PMC4022037; doi:10.1186/1746-4269-10-36)

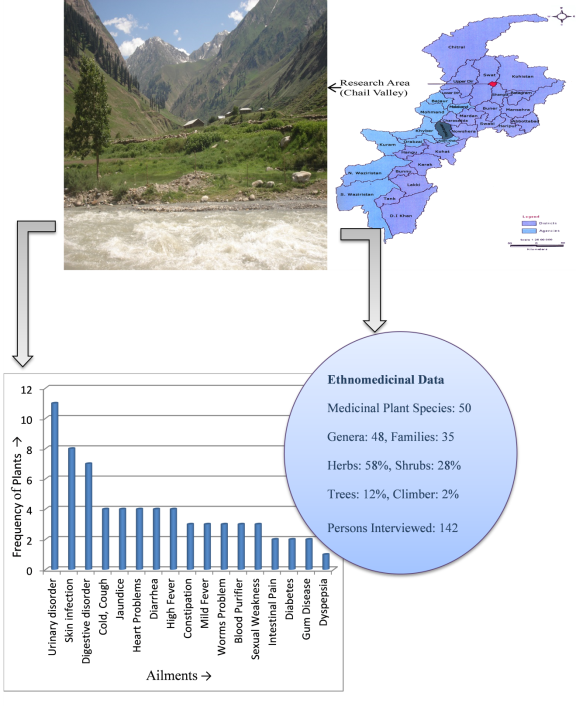

Supplement: Additional file 1 — Graphical abstract of Chail Valley. [file 1746-4269-10-36-S1.docx]
